# Toll-like receptor 5 gene polymorphism is associated with breast cancer susceptibility

**DOI:** 10.18632/oncotarget.20242

**Published:** 2017-08-14

**Authors:** Chen Shuang, Yuan Weiguang, Fu Zhenkun, Huang Yike, Yang Jiankun, Xue Jing, Liu Xinghan, Li Yue, Li Dalin

**Affiliations:** ^1^ Heilongjiang Provincial Key Laboratory for Infection and Immunity, Harbin Medical University and Heilongjiang Academy of Medical Sciences, Harbin, China; ^2^ Department of Immunology, Harbin Medical University, Harbin, China; ^3^ Department of Cancer Immunology, Cancer Institute of Harbin Medical University, Department of Cancer Immunology, Heilongjiang Academy of Medical Sciences, Harbin, China; ^4^ Department of Biochemistry and Molecular Biology, Harbin Medical University, Harbin, China; ^5^ Department of Medical Oncology, Harbin Medical University Cancer Hospital, Harbin, China; ^6^ Department of Breast Surgery, Harbin Medical University Cancer Hospital, Harbin, China

**Keywords:** breast cancer, TLR5, SNP, clinical features

## Abstract

Toll-like receptor 5 (TLR5) plays a fundamental role in immune responses. Recent findings suggest the TLR5 expression level affects cancer progression and development. In the present study, our examination of 256 breast carcinomas specimens revealed that TLR5 is overexpressed in breast carcinomas, and that TLR5 overexpression correlated with lymph node metastasis and cancer grade (p<0.01). In a case-control study, we also analyzed associations between TLR5 single nucleotide polymorphisms (SNPs) and breast cancer risk. Compared were 516 Chinese Han women diagnosed mainly with infiltrative ductal carcinoma and 520 age-matched healthy controls. The nonsense SNP rs5744168 causes truncation of the TLR5 transmembrane signaling domain and was associated with breast cancer risk (p<0.05). However, no statistical association was detected between SNP rs5744168 and any of the clinical parameters tested. Our findings thus indicate that TLR5 SNP rs5744168 is associated with sporadic breast cancer occurrence.

## INTRODUCTION

The etiology of breast cancer is not completely understood, but is thought to result from complex interactions between genetic and environmental factors. For example, chronic inflammation in the vicinity of the tumor microenvironment contributes to disease development and accelerates malignant progression [1]. In addition, variations in some immune regulatory genes appear to drive inter-individual differences in sporadic breast cancer susceptibility [2].

The Toll-like receptor (TLR) family plays a fundamental role in immune systems, particularly in recognition and activation of innate immunity [3]. TLRs recognize corresponding pathogen associated molecular patterns to trigger signal transduction pathways and induce the expressions of immune response molecules, such as inflammatory cytokines [3]. TLR5 recognizes bacterial flagellin from invading bacteria like Salmonella [4, 5]. The reaction between TLR5 and flagellin commonly results in the recruitment of MyD88, and eventually leads to the nuclear localization of NF-κB, which contributes to the initiation of the canonical proinflammatory pathway [5]. Notably, expression of TLR5 is enhanced in many types of carcinomas, including breast cancer [6–9]. Activation of TLR5 triggered by flagellin in breast cancer lines and in mouse xenograft models stimulates production of proinflammatory cytokines and inhibits cell proliferation and anchorage-independent growth [10]. Although no endogenous TLR5 ligands in the tumor environment are yet known, bacterial flagellin may serve as an effective agent for targeted cancer therapy through the activation of TLR5 signaling [11].

Approximately 23% of individuals in the general population carry functional TLR gene polymorphisms that have immunological impact [12, 13]. For example, the single-nucleotide polymorphism (SNP) rs5744168 T allele in TLR5 gene (1174 C>T) encodes a stop codon at codon 392 (TLR5r392x), resulting in truncation of the TLR5 transmembrane signaling domain [13, 14]. This nonsense polymorphism appears to have a relationship with the development of various ailments, including urinary tract infections [15], Legionnaires disease [16], bronchopulmonary dysplasia [17] and cancer [12], among others. Our aim in the present study, therefore, was to clarify the relation between TLR5 coding SNPs and susceptibility to breast cancer in Asian populations. To accomplish this, we took an epidemiologic approach and used tissue microarrays to investigate the relationship between TLR5 and patients’ clinical features. Remarkably, we found SNP rs5744168 and the expression of TLR5 in human breast carcinomas are related to breast cancer occurrence and its clinical features.

## RESULTS

### TLR5 expression is common in breast carcinoma

**Figure 1:**
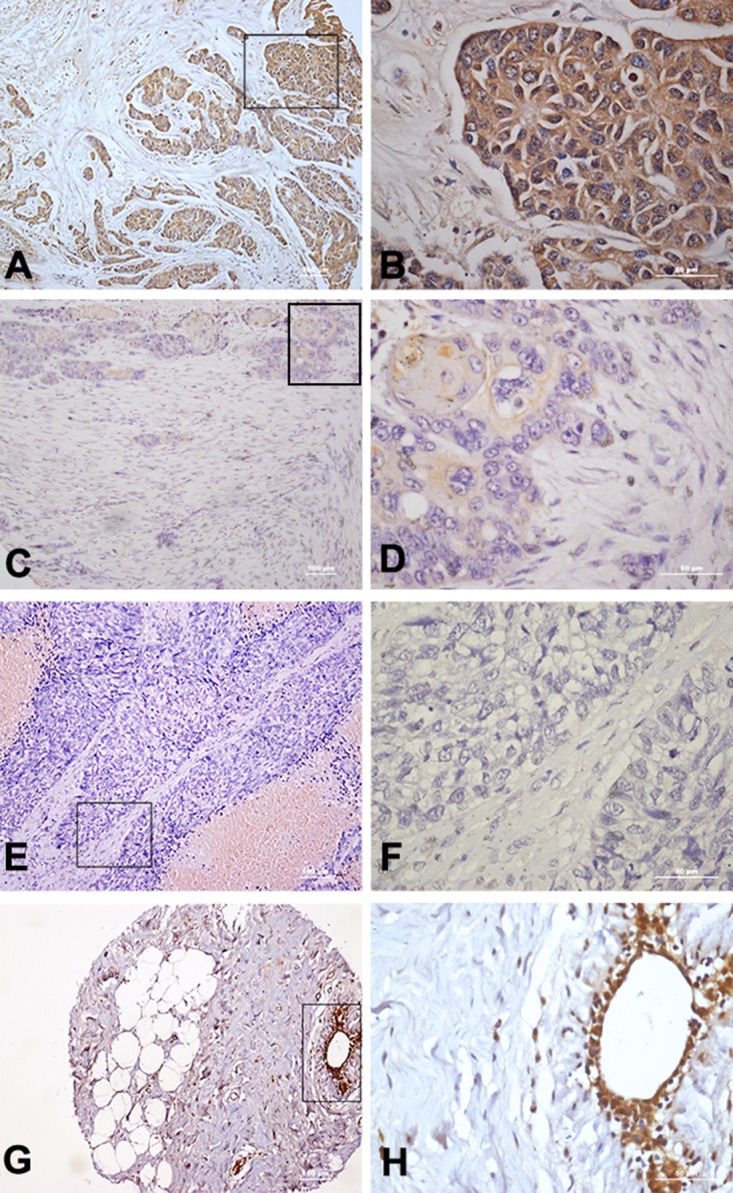
Immunohistochemical staining of TLR5 protein in breast cancer tissues **(A-B)** High expression of TLR5 in breast cancer tissue (10× 40×). **(C-D)** Low expression of TLR5 in breast cancer tissue (10× 40×). **(E-F)** Negative expression of TLR5 in breast cancer tissue (10× 40×). **(G-H)** TLR5 expression on tumor-adjacent breast tissue (10× 40×).

The immunohistochemistry results indicated that TLR5 is often overexpressed in breast cancer. Among the 256 breast cancer tissue samples tested in a microarray, 161 (62.9%) were positive for TLR5, and TLR5 was highly expressed in 66 (25.8%) tumor samples (Figure 1). In tumor-adjacent breast tissues, significant TLR5 expression was also detected in 89.3% breast duct epithelium cells, but not in the fibro-fatty tissue (n=28).

### TLR5 expression in breast carcinoma is associated with lymph node metastasis

The association between the level of TLR5 expression and the clinical features of breast cancer patients were analyzed (Table 1). We found that TLR5 overexpression correlated significantly with lymph node metastasis and tumor grade (p<0.01). However, no difference was observed between TLR5 expression and other clinical features, such as TNM stage and tumor size.

**Table 1:** Correlation between TLR5 expression and clinicopathological parameters

Characteristic	Total no.	Negative expression		Positive expression		Chi-square	p
		n	%	n	%		
Age						1.14	0.285
<=50	148	59	39.9	89	60.1		
>50	108	36	33.3	72	66.7		
TNM stage						2.61	0.456
I	10	5	50.0	5	50.0		
IIA	88	35	39.8	53	60.2		
IIB	85	33	38.8	52	61.2		
III	73	22	30.1	51	69.9		
Tumor size						1.94	0.380
<=5cm	156	55	35.3	101	64.7		
>5cm	53	24	45.3	29	54.7		
Invasion into chest wall	47	16	34.0	31	66.0		
Lymph node metastasis						8.14	**0.004**
Negative	157	69	43.9	88	56.1		
Positive	99	26	26.3	73	73.7		
Grade						14.93	**0.001**
I	47	5	10.6	42	89.4		
II	89	34	38.2	55	61.8		
III	29	14	48.3	15	51.7		
Unknown	91	-	-	-	-		

### A nonsense SNP in TLR5 gene correlates with breast cancer susceptibility in a Chinese Han population

A common nonsense SNP in TLR5 (rs5744168; 392 STOP) results in the truncation of the TLR5, leading to decreased TLR5 signaling. Heterozygous carriers of this SNP have an enhanced susceptibility to some infectious diseases. In the present study, 1036 DNA samples were genotyped SNP for rs5744168 as well as for a missense mutation SNP, rs2072493, which is located in a TLR5 exon and is reportedly associated with decreased survival in colorectal cancer [18]. The distribution of the TLR5 genotypes and alleles in our studied population is shown in Tables 2 and 3. None of these distributions deviated from from Hardy-Weinberg equilibrium (P>0.05). For SNP rs5744168, we observed a higher prevalence of T alleles (p<0.05, OR = 1.215, 95%CI [2.365(1.227-4.561)]) in breast cancer patients than in controls. Statistical significance was also found in the dominant genetic model (gg+ag vs. gg, p=0.011). No statistical significance was also observed between SNP rs2072493 and breast cancer risk in our population.

**Table 2:** Genotype frequencies of TLR5 polymorphisms and their associations with breast cancer risk

SNP	Minor	Major	Case^2^ (%)			Control^3^ (%)			p^4^ for model of inheritance			OR^5^ (95%CI)
	(a^1^)	(A)	AA	Aa	aa	AA	Aa	aa	Additive	Dominant	Recessive	
rs2072493	G	A	292	176	48	270	197	53	0.321	0.132	0.629	0.828(0.649-1.058)
			(56.6)	(34.1)	(9.3)	(51.9)	(37.9)	(10.2)				
rs5744168	T	C	487	28	1	507	13	0	-	0.011	-	2.322(1.193-4.520)
			(94.4)	(5.4)	(0.2)	(97.1)	(2.8)	(0)				

^1^Minor allele ‘a’ and the major ‘A’ are shown in the table. ‘AA’, ‘Aa’, ‘aa’ represent a given variant for each SNP genotyped.

^2^The number of cases in study cohort was 516.

^3^The number of controls in study cohort was 520.

^4^The p values were accessed using Plink and SPSS software under an additive model (AA vs. Aa vs. aa), dominant model (aa+Aa vs. AA), and recessive model (aa vs. Aa+AA) respectively. Significant values (*P*<0.05) are in bold.

^5^Estimated odds ratio (OR) and 95% confidence interval (CI) were assessed under a dominant model (aa+Aa vs. AA).

**Table 3:** Allele frequencies of TLR5 polymorphisms and their associations with breast cancer risk

SNPs	Alleles	NO. (%)		Corrected p	OR (95% CI)
		Cases (n=516)	Controls (n=520)		
rs2072493	A	760 (73.6)	737 (70.9)		Reference
rs2072493	G	272 (26.4)	303 (29.1)	0.336	0.871(0.718-1.055)
rs5744168	C	1002 (97.1)	1027 (98.8)		Reference
rs5744168	T	30 (2.9)	13 (1.3)	**0.021**	2.365(1.227-4.561)

### Relationship between TLR5 SNPs and the clinical features of patients

The clinical features of the 516 breast cancer patients in this case-control study are summarized in Table 4. The correlation between TLR5 SNPs and clinicopathologic features, which included TNM stage, tumor size, lymph node metastasis and the statuses of ER, PR, C-erbB2 and P53, were analyzed. However, no statistical association was detected between SNP rs5744168 and any of the clinical parameters tested.

**Table 4:** Clinicopathologic information from breast cancer patients in the epidemiological study

Clinicopathologic information	Case no. (%)
Tumor Type	
IDC	496 (96.1)
Others	20 (3.9)
Tumor Size (cm)	
Less than 2 =	210 (40.7)
2 to 5 =	228 (44.2)
More than 5	42 (8.1)
Unknown	36 (7.0)
LN metastasis	
Positive	236 (45.7)
Negative	279 (54.1)
Unknown	1 (0.2)
ER	
Negative	192 (37.2)
Positive	292 (56.6)
Unknown	32 (6.2)
PR	
Negative	161 (31.2)
Positive	323 (62.6)
Unknown	32 (6.2)
CerbB-2	
Negative	303 (58.7)
Positive	181 (35.1)
Unknown	32 (6.2)
P53	
Negative	338 (65.5)
Positive	142 (27.5)
Unknown	36 (7.0)

## DISCUSSION

TLRs play a central role in the innate immune response by recognizing pathogen-associated molecular patterns through an extracellular domain and initiating inflammatory signaling pathways through an intracellular domain [3]. Unlike other TLR family members, TLR5 is highly expressed in intestinal epithelial cells and plays key roles in host defense against enterobacterial infections [19]. TLR5 binding with bacterial flagellin can activate NF-kB and trigger proinflammatory and adaptive immune responses to the invading pathogen. Melanie et al. [12] recently demonstrated that microbially driven TLR5 signaling regulates systemic tumor-promoting inflammation and contributes to distal malignant progression. However, the biological importance of TLR5 to tumorigenesis and cancer development is still not completely understood.

Using an epidemiologic approach in the present study, we determined that the nonsense SNP rs5744168 C/T (TLR5r392x) was associated with breast cancer risk in a Chinese Han population. In our cohort, 29 of 516 breast cancer patients carried this stop codon mutation, as compared to 13 of 520 healthy controls. In other studies, SNP rs5744168 was associated with various infectious and autoimmune diseases, including Crohn’s disease [20].

The SNP rs5744168 T allele encodes a stop codon at codon 392 (TLR5r392x) TLR5 gene, leading to truncation of the TLR5 transmembrane signaling domain and abrogation of TLR5 signaling. And because TLR5 usually acts as a homodimer, the TLR5r392x variant may also inhibit assembly and localization of TLR5, thereby impairing immune responses [16]. Carriers of the rs5744168 T genotype were previously observed to exhibit less IL-6 production in response to flagellin than those with the rs5744168 C genotype, and rs5744168 T heterozygotes produced significantly lower levels of proinflammatory cytokines such as TNF-a [21, 22]. We therefore speculated that the reduced ability of the rs5744168 T carriers to induce cytokine production and chronic immune activation results in low immune surveillance function and increases the patient's susceptibility to cancer. Alternatively, rs5744168 may be in linkage disequilibrium with a nearby causative gene that contributes to tumorigenesis. It was also recently reported that TLR5-dependent commensal bacteria drives malignant progression at extra-mucosal locations by increasing systemic IL-6, which drives mobilization of myeloid derived suppressor cells (MDSCs), and that the clinical outcome of cancer patients are thus influenced by TLR5 polymorphism rs5744168 [12]. However, we detected no correlation between TLR5 SNPs and TNM stage, tumor size, lymph node metastasis or the statuses of ER, PR, C-erbB2 and P53.

Another common SNP, rs2072493/N592S (missense mutation) is located in the extracellular domain of TLR5. This SNP was found to be associated with human colorectal cancer survival in a large cohort of Caucasian patients [18], and was also analyzed in this study. In the HT-29 cell line, levels TNF-a mRNA decreased in flagellin-stimulated homozygous N592S or R392X mutant cells, as compared to the wild type [18]. The TLR5 N592S mutation also reportedly affects TNF-a and IFN-r levels in ulcerative colitis patients [23]. This missense mutation SNP was present as a heterozygote frequency of 176+197/1036 (AG) and a homozygote frequency of 292+270/1036 (AA) in our cohort. However, we found no correlation between this SNP and susceptibility of sporadic breast cancer in our Chinese Han population or the patients’ clinicopathologic features (e.g., ER, PR, Her2, P53 status).

The role of TLR expression in breast cancer tissues and its relationship to patients’ clinical features was also investigated in this study. Over 60% of breast carcinomas expressed TLR5 protein in this study. Within breast cancer cells, TLR5 was mainly localized in cytoplasm. TLR5 overexpression correlated negatively with histological grade and positively with lymph node metastasis. Tumor size, lymph node metastasis, hormone receptor expression and tissue grading are commonly used to evaluate the prognosis of patients with breast cancer, among which lymph node metastasis is the most important. These results suggest TLR5 could serve as a biomarker in breast cancer.

In sum, to the best of our knowledge, this study is the first to suggest a link between TLR5 SNPs and breast cancer susceptibility in humans. We consider the combined examination of the epidemiology and TLR5 expression in breast cancer tissues using tissue microarrays an important strength of our study. However, less than 5% of general population harbors the T allele in this Chinese Han cohort. Larger epidemiological studies of ethnically diverse populations, as well as analysis of TLR5 expression in patient-matched tumor samples from the epidemiological study with survival information should be conducted in the future.

## MATERIALS AND METHODS

### Patients’ information

This epidemiological study focused on the relationship between TLR5 SNPs and breast cancer was conducted in the Heilongjiang Province in northeastern China. This research was approved by the institutional ethical committees of the Third Affiliated Hospital of Harbin Medical University and the Harbin Medical University. All of the volunteers gave informed written consent. Peripheral blood samples were collected from 516 female breast cancer patients referred to the Third Affiliated Hospital of Harbin Medical University. The study participants ranged from 20 to 77 years of age (mean: 49.6 years) and were all diagnosed based on surgical and pathological symptoms. Healthy controls with no history of personal or familial malignancy or autoimmune disorders were frequency matched to the patients based on age and residence in the same Chinese area. The survival data were not successfully collected from this cohort. However, clinical features such as tumor size, lymph node metastasis, human epidermal growth factor receptor 2 (C-erbB2), estrogen receptor (ER), progesterone receptor (PR) and tumor protein 53 (P53) statuses, which have significant overall association with patient prognosis and outcome, were obtained from the medical files. The tumor samples used for immunohistochemical detection including 170 invasive ductal carcinomas, 81 invasive lobular carcinomas, and 5 other (medullary carcinoma, mucinous carcinoma, and Squamous cell carcinoma) pathology diagnosis types obtained from the US Biomax Company in the form of tissue microarrays. No prognostic information was available from the tissue microarrays we analyzed.

### DNA extraction and genotyping

Genomic DNA was extracted from frozen whole blood using a Universal Genomic DNA Extraction Kit, version 3.0 (TaKaRa, Japan) according to the manufacturer’s protocol. Genotyping was performed using polymerase chain reaction restriction fragment length polymorphism (PCR-RFLP) assays (Supplementary Figure 1). The polymorphic region was amplified by PCR using a T-Gradient Thermoblock PCR System (Biometra, Germany) in a 25-μl reaction mixture containing about 0.3 μg of genomic DNA, 2.5 μl of 10x PCR buffer (Mg^2+^ plus), 2 μl of dNTPs, 0.5 μl of TaqDNA polymerase (TaKaRa, Japan), and 1 μl of each primer (10 μM). Primers, annealing temperatures and restriction enzymes for PCR-RFLP genotyping are listed with the supplementary information (Supplementary Table 1). After PCR-RFLP analysis, random purified PCR products were sequenced directly using an ABI-3730xp automatic DNA sequencer (Applied Biosystems) to confirm the accuracy of the genotyping results (Supplementary Figure 2).

### Immunohistochemical staining

Immunohistochemical detection of TLR5 protein was conducted on breast carcinoma and pericarcinomatous tissue samples from female breast cancer patients with clear pathologic diagnoses. Paraffin-embedded samples were stained with purified rabbit anti-human TLR5 primary IgG (1:100 dilution; Proteintech). The slides were counterstained with hematoxylin. Images were obtained using a Nikon Eclipse 80i microscope. The staining of each sample was scored independently by two pathologists blinded to the clinicopathological findings.

TLR5 expression was assessed by evaluating the proportion and intensity of staining, which was considered as representative of the average in a 40× magnification field. Briefly, the proportion of positively stained tumor cells in a field was scored as follows: 0, none; 1, <10%; 2, 10% to 50%; and 3, >50%. The staining intensity in a field was scored as follows: 0, no staining; 1, weak staining, appearing as light yellow; 2, moderate staining, appearing as yellowish-brown; and 3, strong staining, appearing as brown. The staining index (SI) was calculated as: (averaged staining intensity score) × (proportion score). An SI >1 was deemed to indicate TLR5 positivity. An SI of 4 (a cut-off point) was used to distinguish between low (≤4) and high (>4) TLR5 expression.

### Statistical analysis

Deviation from Hardy-Weinberg equilibrium (HWE) was determined using a goodness-of-fit chi-squared test to compare the observed genotype frequencies with the expected frequencies from the healthy controls. The polymorphisms were excluded if they deviated from the HWE or if missing data comprised more than 10% of the total data. Using different models of inheritance (additive, dominant, recessive), the genotype frequencies of were analyzed using the chi-squared test and Fisher’s exact test. Estimated odds ratios (ORs) and 95% confidence interval (CIs) were assessed with Plink and SPSS software. To determine the significance with corrections for multiple testing biases, we ran 10,000 permutations using Haploview to determine the corrected p value. All data were analyzed using SPSS (version 17.0), Plink (version 1.07) (http://pngu.mgh.harvard.edu/~purcell/plink), and Haploview (version 4.1) (http://www.broad.mit.edu/mpg/haploview/). The threshold for significance was p<0.05, and the relative risks associated with haplotypes were estimated as ORs with 95% CIs.

## SUPPLEMENTARY MATERIALS FIGURES AND TABLE



## Abbreviation

IDC: infiltrative ductal carcinoma
