# Supplementary material for: Toll-like receptor 5 gene polymorphism is associated with breast cancer susceptibility
Source: Oncotarget. 2017 Aug 14;8(51):88622–9. doi: 10.18632/oncotarget.20242 (PMC5687632; doi:10.18632/oncotarget.20242)
Supplement: Supplementary file 1 [file oncotarget-08-88622-s001.pdf]

Toll-like receptor 5 gene polymorphism is associated with breast cancer susceptibility

SUPPLEMENTARY MATERIALS

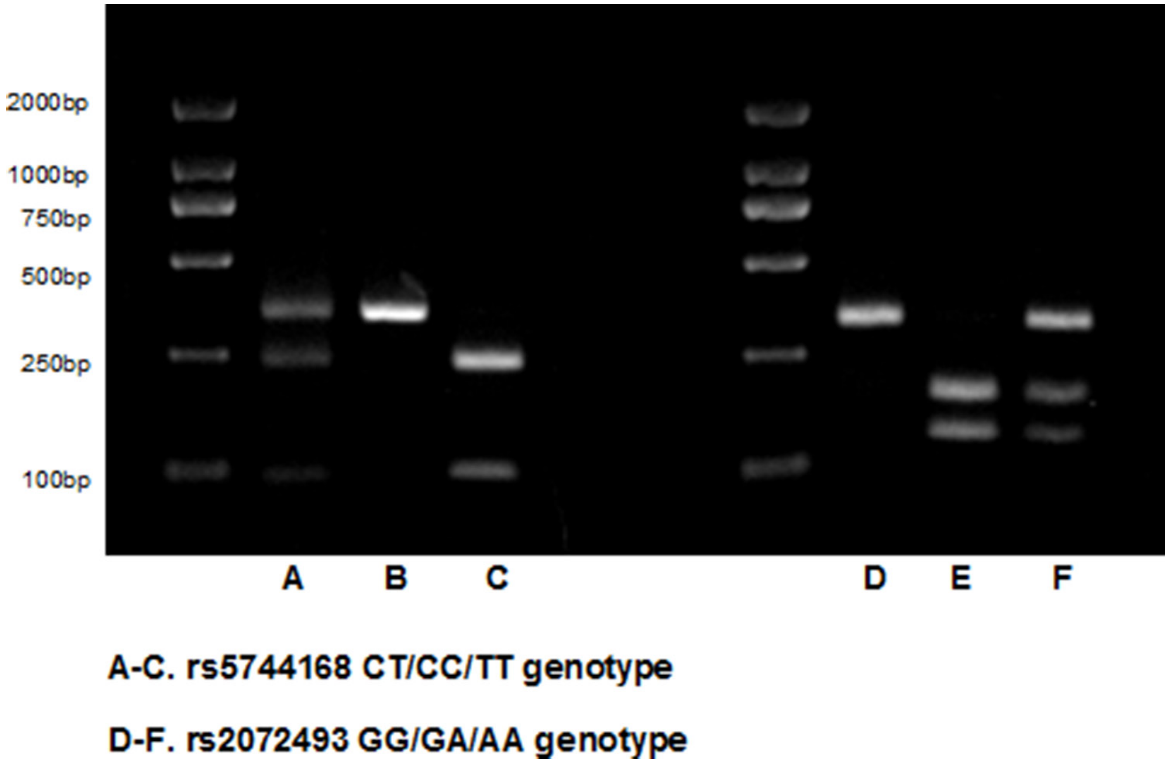

Supplementary Figure 1: PCR-RFLP results for rs2072493 and rs5744168.

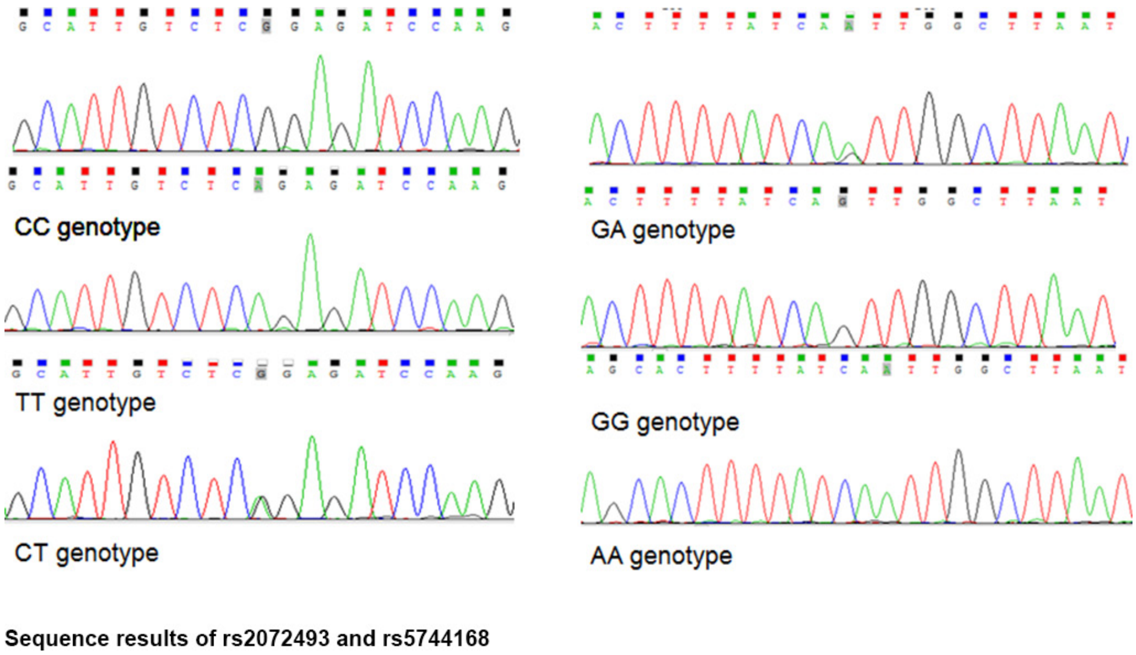

Supplementary Figure 2: Sequencing results for rs2072493 and rs5744168.

Supplementary Table 1: Supplement information for PCR-RFLP genotyping

SNP	Primer	Annealing temperature	Restriction enzyme
rs2072493	5'-GACTAAGCCTCAACTCCAACA-3'	58.0	MfeI
	5'-GACTTCCTCTTCATCACAACC-3'		
rs5744168	5'-GGTAGCCTACATTGATTTGC-3'	56.8	DdeI
	5'-GGATTCTCTGAAGGGGTTTGAT-3'		
